# Supplementary material for: A 69 kb Deletion in chr19q13.42 including PRPF31 Gene in a Chinese Family Affected with Autosomal Dominant Retinitis Pigmentosa
Source: J Clin Med. 2022 Nov 11;11(22):6682. doi: 10.3390/jcm11226682 (PMC9695658; doi:10.3390/jcm11226682)
Supplement: Supplementary file 1 [file jcm-11-06682-s001.zip › Supplementary materials.pdf]

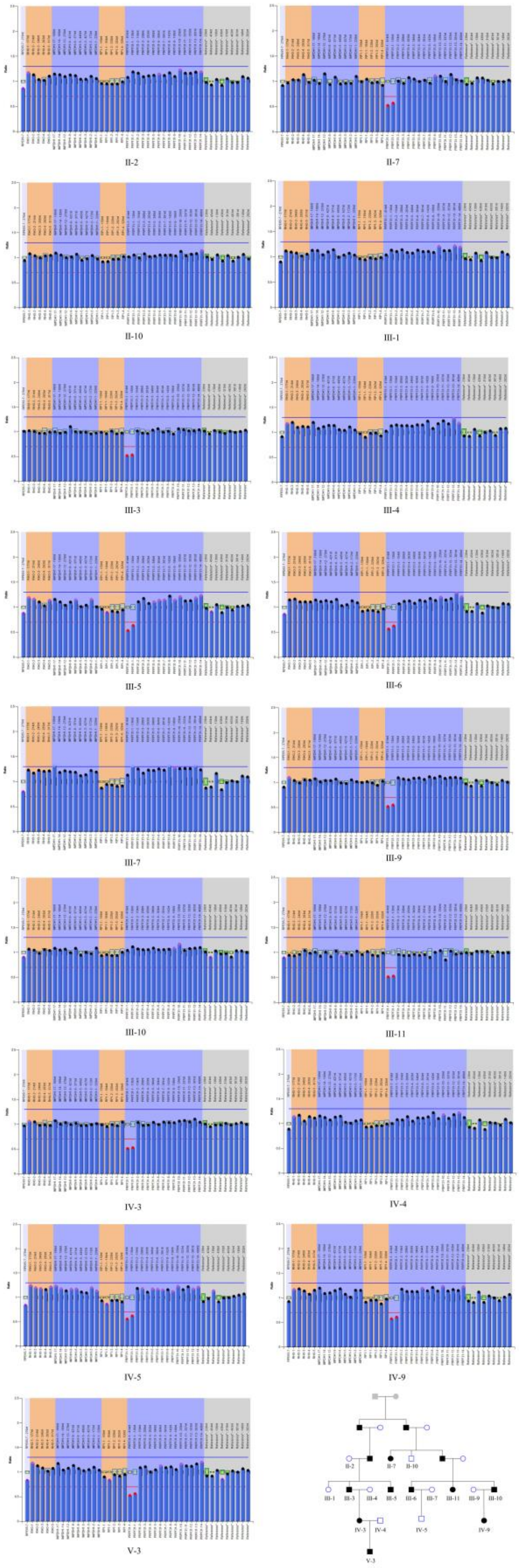

Figure S1. The results of multiplex ligation-dependent probe amplification (MLPA).

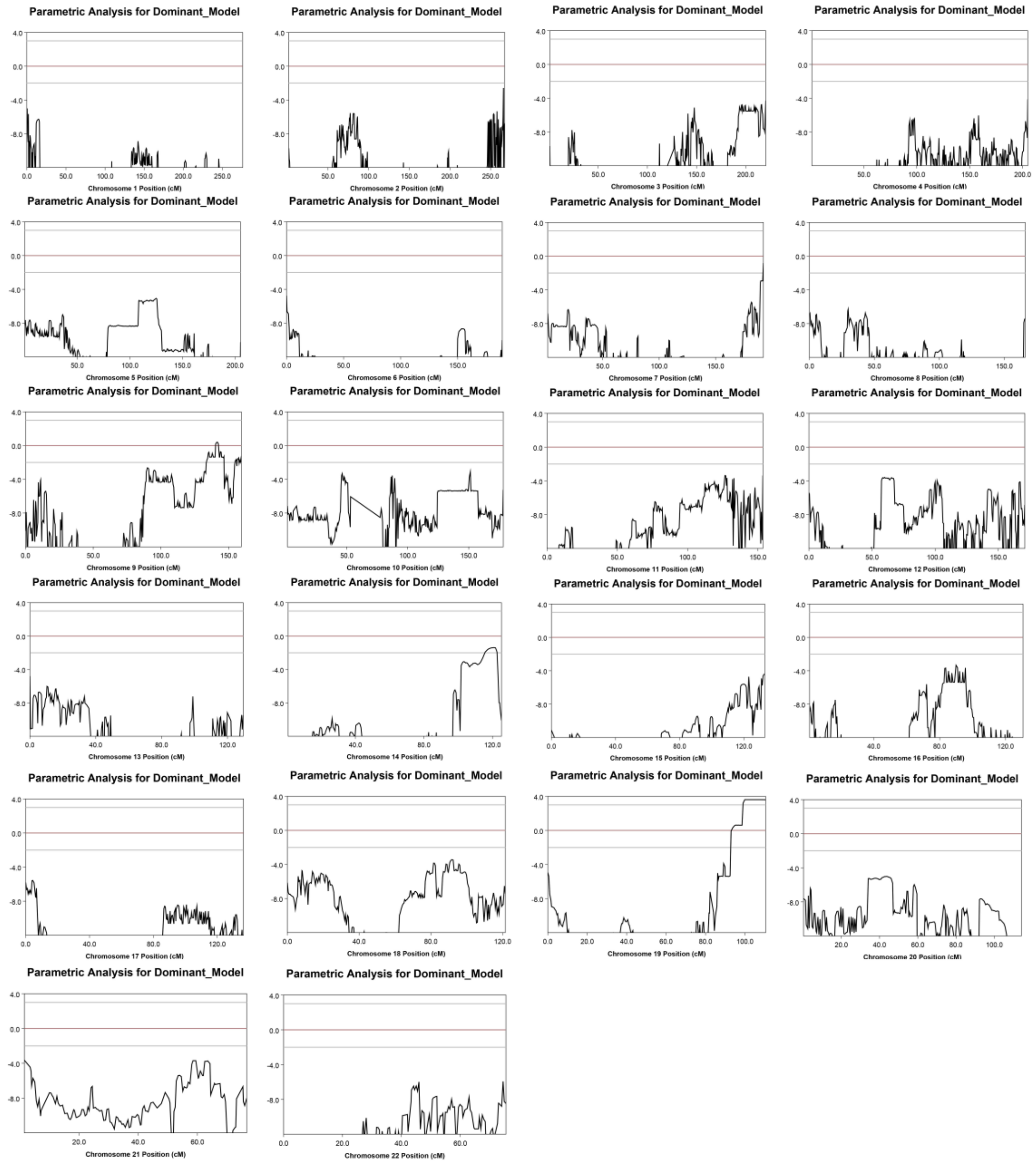

Figure S2. The parametric analysis of autosomal 1-22.

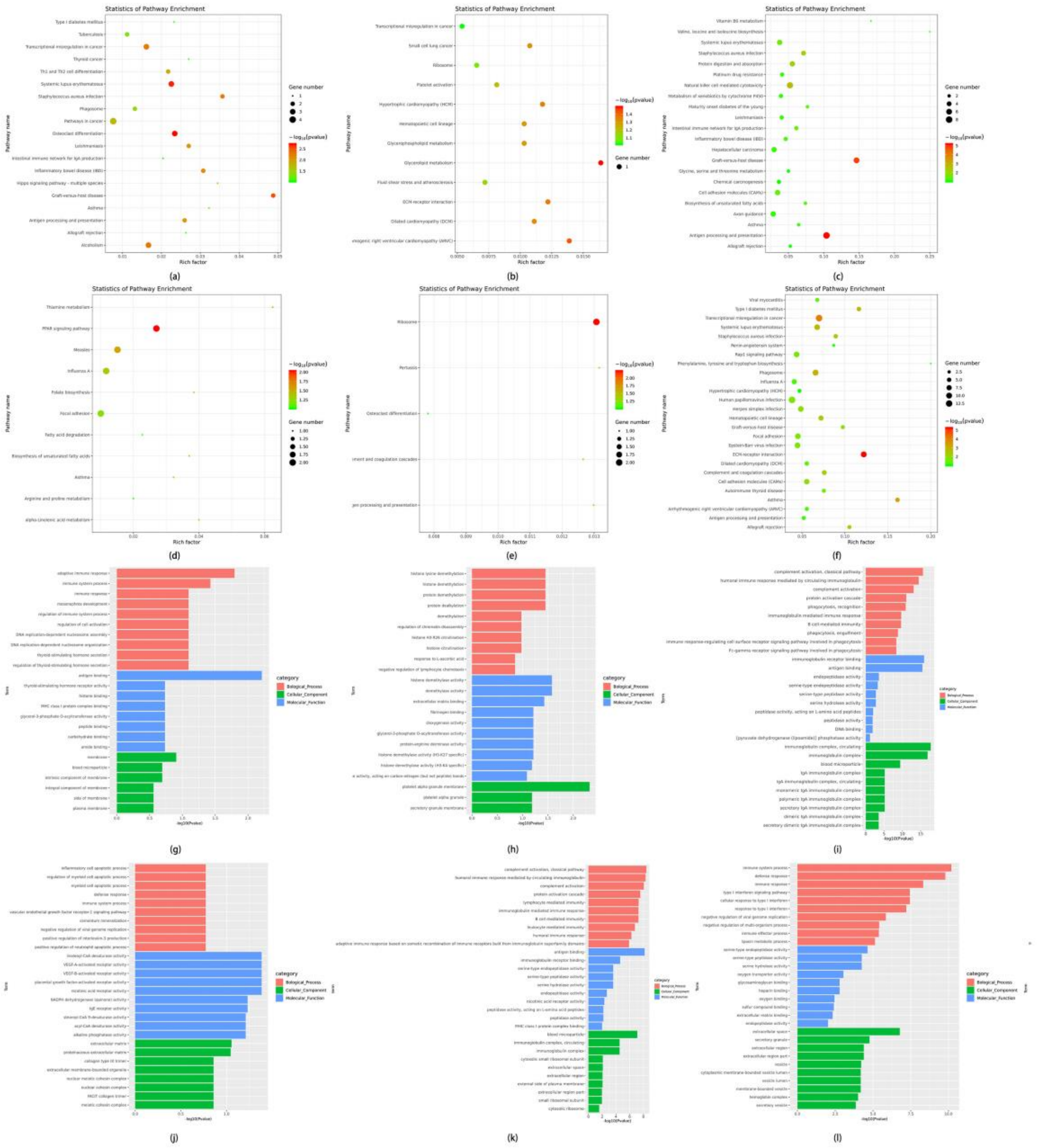

Figure S3. Gene oncology (GO) term and Kyoto encyclopaedia of genes and genomes (KEGG) function enrichment of differential expressive genes (DEGs) analysis. (a-c) and (g-i) represent the up-regulated gene and (d-f) and (j-l) represent the down-regulated genes. The left is the Ⅲ-7 vs Ⅲ-6, the middle is Ⅳ-5+Ⅳ-7 vs Ⅲ-7 and the right row is Ⅳ-5+Ⅳ-7 vs Ⅲ-6.

Table S1. Panel of next generation sequencing for fundus diseases

Genes list							
RGS9BP	B3GLCT	PANK2	NPHP3	VPS13B	CLDN19	RPIA	LRPPRC
ALG2	SBF2	COL18A1	SOST	NDUFS7	FKRP	TEAD1	SALL4
EIF2B3	TMCO1	HMX1	PEX26	CHD7	AHI1	ZNF644	TMEM216
AAAS	EIF2B4	SACS	EIF2B2	OSTM1	POMT1	NDUFV1	SOX10
PHYH	TCIRG1	ALG3	EPM2A	HCCS	SLC12A6	CTNS	KRIT1
LARGE1	SLC24A1	NDUFS3	LRP2	HSD17B10	GPC3	COX15	BCS1L
ATIC	ACOX1	PAX2	EIF2B5	HESX1	RGS9	PDHX	SURF1
SH3BP2	PRPS1	TNFRSF11B	NDUFS8	NDUFS4	LAMB2	ERBB3	OPN1SW
IGBP1	EIF2B1	CLCN7	PRSS56	CISD2	TRIM37	PTGS1	GABRB3
CYP27A1	TSC2	PLP1	ARSA	APOC2	PEX1	NOTCH3	NEU1
ALDH3A2	TSC1	ATXN7	ATXN1	PEX2	PTEN	PMM2	PLOD1
PEX7	PEX6	PEX12	NAGA	MTTP	MITF	JAG1	IDS
HSD11B2	SHH	HADHA	GP1BA	GALC	G6PC	FGFR2	CP
ERCC8	LYST	ASPA	TIMM8A	CAPN5	IMPG1	RCBTB1	EPHA2
ARHGEF18	REEP6	AGBL5	HGSNAT	IFT172	CCDC28B	NEK2	IFT74
IFT27	POC1B	SLC19A2	NBAS	MFF	SLC25A46	ACO2	MECR
TBCE	UCHL1	RTN4IP1	PMPCA	ISCA2	ATP1A3	YME1L1	KLC2
CTNNB1	COL4A4	COL4A3	WDPCP	FLVCR1	CYP4V2	RP9	CERKL
CRB1	EYS	RD3	LCA5	RP1L1	USH1G	CACNA2D4	OTX2
BBS12	RDH12	BBS5	CABP4	ZNF513	TTC8	KCNV2	COL9A1
CDHR1	PCDH15	BBS4	RAX2	TMEM126A	ARL6	HMCN1	BBS2
MFRP	PITPNM3	OPA3	CEP290	BBS10	BBS1	ELOVL4	NYX
SEMA4A	CDH23	RPGRIP1	CNNM4	OPN1LW	INPP5E	CNGB3	TMX3
MKKS	SPATA7	BBS7	CLN6	POMGNT1	C1QTNF5	PRPF31	OPA1
RPGRIP1L	ALMS1	NPHP4	RIMS1	AIPL1	NR2E3	SNRNP200	POMT2
PRPF6	FSCN2	RAB3GAP1	FZD4	USH2A	RP2	IDH3B	RBP4
SDCCAG8	CLN5	PRPF8	MERTK	FBLN5	RP1	PDE6C	NTF4
NRL	PROM1	WFS1	TOPORS	TSPAN12	USH1C	GNAT2	CACNA1F
UNC119	LRAT	PRPF3	VCAN	SDHA	EFEMP1	ADAM9	IKBKG
TULP1	SPG7	SOX2	GRK1	RGR	RDH5	RBP3	PDE6G

TRPM1	GUCA1B	COL11A1	COL2A1	CDH3	CNGA3	CNGB1	DHDDS
MAK	KCNJ13	TRIM32	CC2D2A	PRCD	BBS9	KLHL7	C2orf71
IQCB1	OPTN	IMPDH1	GRM6	CA4	CRX	VHL	SAG
RHO	HEXB	HEXA	OPN1MW	PDE6A	LTBP2	GUCA1A	CHM
TYR	TTPA	TIMP3	ABCA4	RPE65	RPGR	ROM1	RLBP1
PRPH2	RB1	PPT1	PDE6B	PAX6	OAT	GPR143	NPHP1
NDP	MYOC	MYO7A	GUCY2D	GNAT1	FBN1	CYP1B1	CNGA1
CLN3	OFD1	FAM161A	BEST1	IMPG2	CLRN1	C8orf37	SLC7A14
KIZ	PRPF4	ZNF408	ARL2BP	ITM2B	MFSD8	RAB28	TTLL5
PCYT1A	PDE6H	RS1	GPR179	LRIT3	CFI	SIX6	NMNAT1
GDF6	FKTN	GMPPB	SLC45A2	TYRP1	OCA2	SLC24A5	LRP5
ASB10	WDR36	FOXC1	POLG	SLC25A4	TWNK	POLG2	RRM2B
SALL2	SCO2	PRIMPOL	SLITRK6	RAX	ALDH1A3	STRA6	ABCB6
SMOC1	FRMD7	ABHD12	ADAMTS18	ADAMTSL4	COL11A2	ADGRV1	CIB2
PDZD7	MKS1	TMEM67	LZTFL1	BBIP1	CASK	NR2F1	OCRL
TGFBR1	TGFBR2	TPP1	DNAJC5	CLN8	CTSD	ATM	MRE11
C12orf65	CACNA1A	PRKCG	HGF	LOXHD1	DNM1L	FAM126A	MFN2
MPDZ	RAD51	SCN2A	SLC9A6	SOD1	TK2	TTC21B	C5AR2
NXNL1	OPTC	NR2E1	DOCK9	OLFM2	KIF11	GNPTG	SRPX
EMC1	MYO15A	DHX38	NEUROD1	OR2W3	DRAM2	APOE	CFHR1
CFHR3	CFH	ERCC6	HTRA1	C3	CST3	CX3CR1	C2
C9	FBN2	ATF6	NAA10	BCOR	BMP4	VAX1	RARB
HMGB3	MAB21L2	TENM3	TMEM98	HDAC6	GLI2	YAP1	C12orf57
ACTB	PIGL	SRD5A3	TFAP2A	PITX2	ELP4	LRPAP1	SLC39A5
P3H2	ANTXR1	WDR73	SLC38A8	TUBA8	PNPLA6	RDH11	TUB
LRMDA	TUBGCP4	TUBGCP6	PLA2G5	POMGNT2	TMEM5	POMK	CHN1
TREX1	SLC16A12	ATOH7	WHRN	HARS	WDR19	ARL13B	ATP13A2
CTSF	KCTD7	GLB1	IGFBP7	CTC1	ACBD5	C21orf2	CEP41
CSPP1	KIF7	TCTN1	TCTN3	TMEM138	TMEM231	TMEM237	ZNF423
CNTNAP2	COL4A1	FOXE3	LMX1B	LOXL1	TBK1	VSX2	GDF3
GJA1	SIX3	CDKN2B-AS1	tRNA-His	tRNA-Lys	tRNA-Leu1	tRNA-Pro	tRNA-Ser1
tRNA-Ser2	tRNA-Val	tRNA-Trp	ND4	ND4L	COX3	ATP6	CYTB

	ND6	ND5	ND3	ND2	COX1	ND1

Table S2. The number of mutations in *PRPF31* and related phenotypes.

Phenotype	Missense /nonsense	Splicing	Small deletions	Small insertions	Small indels	Gross deletions	Gross insertions /duplications	Complex rearrangements	Repeat variants	Regulatory
Retinitis Pigmentosa	62	34	56	25	4	20	7	3	0	0
Retinal disease	5	0	1	0	0	0	0	0	0	0
Retinal Dystrophy	4	2	2	0	0	0	0	0	0	0
Retinal Degeneration	1	1	5	2	0	0	0	0	0	0
Leber congenital amaurosis	1	0	0	0	0	0	0	0	0	0
Development disorder	0	1	0	0	0	0	0	0	0	0
Total	73	38	64	27	4	20	7	3	0	0

Table S3. Expression level of genes in deletion region and linkage region

Gene	Full_name_of gene	Map_location	Type_of_gene	Ⅲ-6	Ⅳ-5	Ⅳ-7	Ⅲ-7
PRPF31	pre-mRNA processing factor 31	19q13.42	protein-coding	16.44437192	14.94919772	15.70212802	31.06400293
VSTM1	V-set and transmembrane domain containing 1	19q13.42	protein-coding	6.541407613	1.293074065	2.107323236	5.407333238
TARM1	T cell-interacting, activating receptor on myeloid cells 1	19q13.42	protein-coding	0.242274356	0.094615175	0	0
NDUFA3	NADH:ubiquinone oxidoreductase subunit A3	19q13.42	protein-coding	9.781827125	7.632290821	6.527562219	14.47588169
OSCAR	osteoclast associated, immunoglobulin-like receptor	19q13.42	protein-coding	34.61494862	13.05689421	18.58042317	45.39906865
TFPT	TCF3 fusion partner	19q13.42	protein-coding	5.542025894	3.879222194	3.186683918	6.477534608
TNNT1	troponin T1, slow skeletal type	19q13.42	protein-coding	9.964	0.978	1.362	0.929
LILRB3	leukocyte immunoglobulin like receptor B3	19q13.42	protein-coding	234.34	85.469	108.81	113.047
LAIR2	leukocyte associated immunoglobulin like receptor 2	19q13.42	protein-coding	2.332	0.315	0.206	3.267
LILRP1	leukocyte immunoglobulin-like receptor pseudogene 1	19q13.42	pseudo	0.03	0.946	0.822	2.028
RFPL4A	ret finger protein like 4A	19q13.42	protein-coding	0	0.757	0.668	1.577
KIR2DL3	killer cell immunoglobulin like receptor, two Ig domains and long cytoplasmic tail 3	19q13.42	protein-coding	1.121	4.163	1.619	6.055
KIR2DL1	killer cell immunoglobulin like receptor, two Ig domains and long cytoplasmic tail 1	19q13.42	protein-coding	0.03	4.731	0.642	1.493

KIR3DL2	killer cell immunoglobulin like receptor, three Ig domains and long cytoplasmic tail 2	19q13.42	protein-coding	1.393	3.059	1.336	5.689
CACNG6	calcium voltage-gated channel auxiliary subunit gamma 6	19q13.42	protein-coding	2.574	0.442	0.899	0.591
TMC4	transmembrane channel like 4	19q13.42	protein-coding	1.121	2.113	2.853	3.605
KIR3DL1	killer cell immunoglobulin like receptor, three Ig domains and long cytoplasmic tail 1	19q13.42	protein-coding	0.454	4.731	0.642	1.971
TPM3P9	tropomyosin 3 pseudogene 9	19q13.42	pseudo	10.69	9.083	9.226	4.591
ZNF761	zinc finger protein 761	19q13.42	protein-coding	4.755	8.484	8.506	10.702
ZNF550	zinc finger protein 550	19q13.42	protein-coding	4.149	8.736	8.404	9.238

Table S4. Expression level of genes related to pre-mRNA splicing and function of genes in linkage region.

Gene	Full_name of gene	Map_location	Type_of_gene	Ⅲ-6	Ⅳ-5	Ⅳ-7	Ⅲ-7
SNRPD3	small nuclear ribonucleoprotein D3 polypeptide	22q11.23	protein-coding	32.07106788	32.10608287	28.57735901	32.33134665
PHF5A	PHD finger protein 5A	22q13.2	protein-coding	13.65821682	13.21458617	13.10652257	14.39139211
PRPF6	pre-mRNA processing factor 6	20q13.33	protein-coding	78.07291124	74.46214309	74.96416927	68.35207172
SF3A2	splicing factor 3a subunit 2	19p13.3	protein-coding	53.72433845	46.86605025	45.89852805	43.48397146
PRPF3	pre-mRNA processing factor 3	1q21.2	protein-coding	41.73175783	45.06836191	42.68614506	43.2868291
LSM8	LSM8 homolog, U6 small nuclear RNA associated	7q31.31	protein-coding	10.4480816	13.93996918	15.93341959	15.15179834
SNRPA1	small nuclear ribonucleoprotein polypeptide A'	15q26.3	protein-coding	15.02101007	15.23304325	14.77696172	15.99669416
TXNL4A	thioredoxin like 4A	18q23	protein-coding	26.28676763	22.83379568	24.64540224	24.38932596
SNRNP200	small nuclear ribonucleoprotein U5 subunit 200	2q11.2	protein-coding	204.6006937	248.8694499	229.1585524	226.7700377
SART1	squamous cell carcinoma antigen recognized by T-cells 1	11q13.1	protein-coding	86.88564094	67.05062101	77.27708501	66.26799536
SNU13	small nuclear ribonucleoprotein 13	22q13.2	protein-coding	50.78676189	44.59528603	42.45485349	50.58109633
SNRNP27	small nuclear ribonucleoprotein U4/U6.U5 subunit 27	2p13.3	protein-coding	12.29542357	12.77304869	13.05512444	12.16649979
PRPF4	pre-mRNA processing factor 4	9q32	protein-coding	17.92830235	18.98611188	17.68095593	17.82730177
USP39	ubiquitin specific peptidase 39	2p11.2	protein-coding	38.24906396	30.15070258	37.62342948	33.65501677

SART1	squamous cell carcinoma antigen recognized by T-cells 1	11q13.1	protein-coding	86.88564094	67.05062101	77.27708501	66.26799536
GSTM1	glutathione S-transferase mu 1	1p13.3	protein-coding	0	7.664	9.586	7.745
ERAP2	endoplasmic reticulum aminopeptidase 2	5q15	protein-coding	13.113	80.833	87.762	149.321
TNNT1	troponin T1, slow skeletal type	19q13.42	protein-coding	9.964	0.978	1.362	0.929
SLIT1	slit guidance ligand 1	10q24.1	protein-coding	1.09	0	0	0
MARCKS	myristoylated alanine rich protein kinase C substrate	6q21	protein-coding	120.955	67.177	58.311	54.439
IRF7	interferon regulatory factor 7	11p15.5	protein-coding	186.309	56.012	88.019	101.247
GSTM3	glutathione S-transferase mu 3	1p13.3	protein-coding	1.181	5.488	3.032	6.083
